# Pustular psoriasis flare following COVID-19 infection: a case report and literature review

**DOI:** 10.3389/fimmu.2026.1740000

**Published:** 2026-03-17

**Authors:** Eri Ohta, Etsuko Okada, Yu Sawada

**Affiliations:** 1 Department of Dermatology, Takasaki General Medical Center, Takasaki, Japan; 2 Department of Dermatology, University of Occupational and Environmental Health, Kitakyushu, Japan

**Keywords:** case report, COVID-19, COVID-19 vaccine, generalized pustular psoriasis, literature review

## Abstract

Generalized pustular psoriasis (GPP) is a rare, potentially life-threatening inflammatory disease characterized by neutrophilic pustules and systemic inflammation. We report a case of severe GPP triggered by SARS-CoV-2 infection in a 46-year-old woman with a long history of psoriasis. Eleven days after recovery from COVID-19 pneumonia, she developed widespread pustules and fever. Histopathology revealed subcorneal spongiform pustules and dermal neutrophilic infiltration consistent with GPP. Systemic corticosteroids followed by etretinate and deucravacitinib achieved complete remission. A literature review identified 11 infection- and 10 vaccine-related GPP cases. Compared with vaccine-associated cases, infection-related flares showed longer latency and higher corticosteroid use. Mechanistically, both SARS-CoV-2 infection and vaccination may be associated with IL-36 axis activation, potentially via spike protein–driven, Toll-like receptor–mediated innate immune signaling. This case highlights that distinct immune kinetics may underlie infection- and vaccine-related GPP, while supporting a putative role of IL-36–driven inflammation in COVID-19–associated disease exacerbation.

## Introduction

Generalized pustular psoriasis (GPP) is a rare, potentially life-threatening inflammatory skin disease characterized by widespread sterile pustules, fever, and systemic symptoms (1). Exacerbations can be triggered by various factors, including infections, pregnancy, drug exposure, and corticosteroid withdrawal, yet in many cases, the precipitating event remains elusive (2).

Since the emergence of the COVID-19 pandemic, numerous immune-mediated skin diseases have been reported in association with either SARS-CoV-2 infection or vaccination (3). Among them, several cases of GPP flares have drawn particular attention (4), suggesting that both viral infection and immune stimulation may perturb cytokine homeostasis in susceptible individuals. However, the precise mechanisms linking SARS-CoV-2–related immune activation to GPP remain unclear.

Here, we report a case of severe GPP triggered by COVID-19 infection in a patient with a long-standing history of psoriasis. Through a review of published cases, we compare the clinical and immunologic features of infection- and vaccine-associated GPP and discuss potential mechanisms underlying their distinct temporal and inflammatory patterns.

## Case description

### Patient information

A 46-year-old Japanese woman presented with high fever, fatigue, and rapidly spreading erythematous eruptions with pustules involving the trunk and extremities. She had a long-standing history of psoriasis, initially diagnosed as scalp psoriasis vulgaris at the age of 12, which progressed to GPP and psoriatic arthritis by the age of 20. Previous treatments included cyclosporine, adalimumab, secukinumab, and apremilast, all of which resulted in partial remission. She discontinued treatment on her own, after which her skin condition gradually worsened over the following two years. During this period, at the age of 45, she experienced an acute myocardial infarction requiring hospitalization at our institution, after which she resumed regular dermatologic follow-up.

### Clinical findings

**Figure 1:**
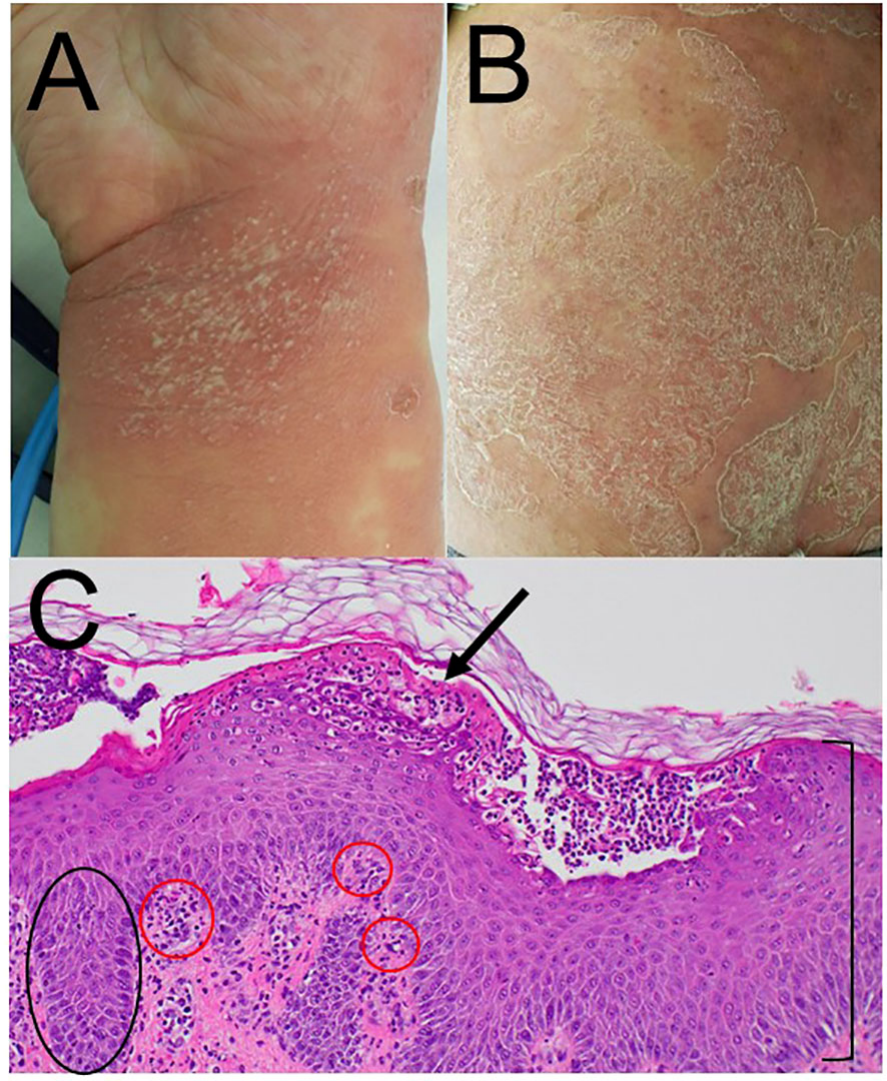
Clinical manifestation and histological examination. **(A)** Erythematous plaques with numerous non-follicular pustules coalescing into lakes of pus on the right wrist. **(B)** Erythematous pustular eruption with scaling on the left buttock. **(C)** Histopathological examination of a pustular lesion showing elongation of rete ridges (indicated by black circles) and acanthosis of the epidermis (indicated by a black parenthesis-shaped mark), neutrophilic infiltration in the epidermis and upper dermis (red circles), and subcorneal spongiform pustules (Kogoj’s spongiform abscesses) (black arrow) (hematoxylin and eosin stain).

At the age of 46, the patient developed COVID-19 pneumonia confirmed by polymerase chain reaction testing and was admitted to the department of internal medicine. She received ceftriaxone and amoxicillin/clavulanate for suspected bacterial co-infection and was discharged after 6 days. However, 11 days after the onset of COVID-19, she developed a high-grade fever (38.2 °C), marked fatigue, and widespread erythematous plaques with superficial pustules and scaling over the trunk and extremities, prompting readmission to our dermatology department. Physical examination revealed diffuse erythematous plaques with numerous non-follicular pustules coalescing into lakes of pus, without mucosal involvement (**Figures 1A, B**).

### Diagnostic assessment

Laboratory investigations demonstrated severe systemic inflammation and neutrophilia, with a white blood cell count of 29.6 × 10³/µL (neutrophils 91.1%) and a C-reactive protein level of 18.85 mg/dL. Serum albumin was decreased to 3.4 g/dL, total bilirubin was elevated to 1.97 mg/dL, and mild renal dysfunction was observed (creatinine 1.13 mg/dL; estimated glomerular filtration rate 41.8 mL/min/1.73 m²). According to the Japanese severity scoring system for GPP (2010), the total score was 14, indicating severe disease. Histopathological examination of a skin biopsy obtained from an active pustular lesion revealed acanthosis with elongated rete ridges, neutrophilic infiltration in the upper dermis, and subcorneal spongiform pustules (Kogoj’s spongiform abscesses) (**Figure 1C**). Gram and periodic acid–Schiff staining revealed no bacterial or fungal elements. Based on these clinical, laboratory, and histopathological findings, a diagnosis of GPP flare was established.

### Therapeutic intervention

**Figure 2:**
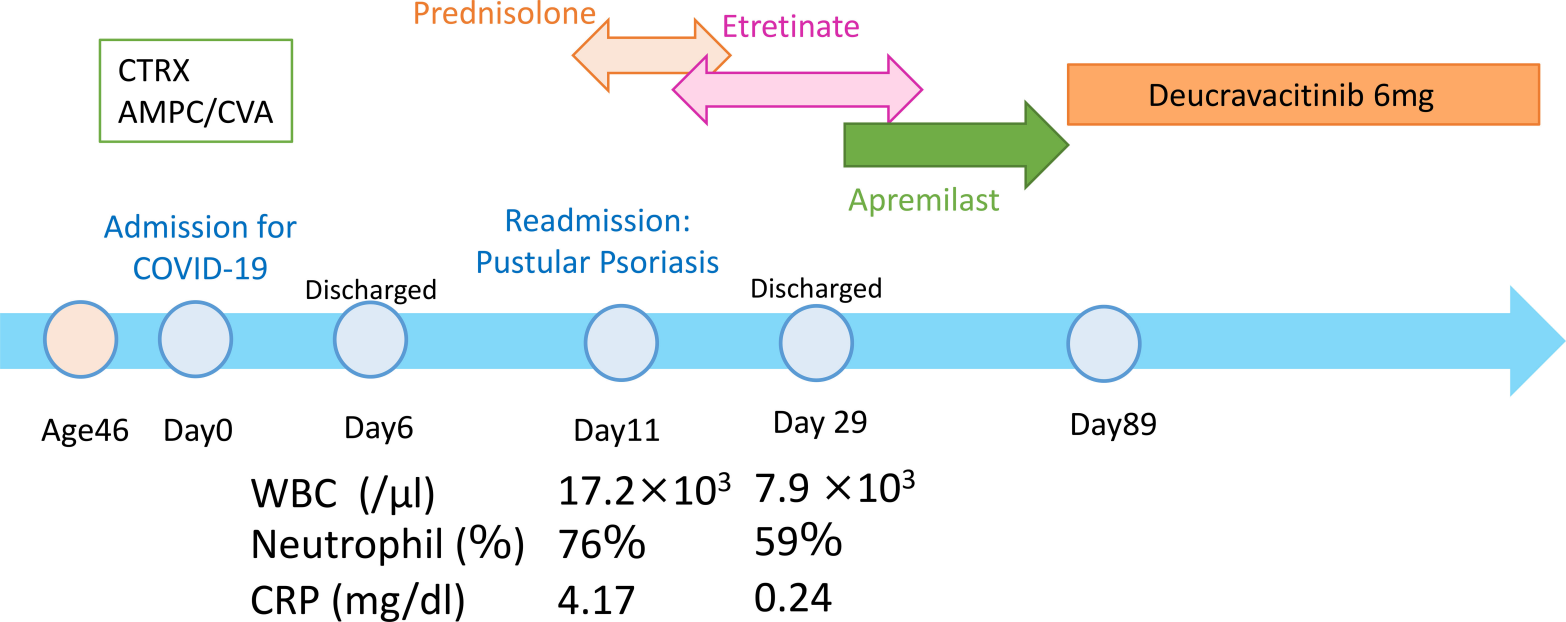
Clinical course and treatment timeline of a patient with pustular psoriasis triggered by SARS-CoV-2 infection. After COVID-19 pneumonia, a severe pustular flare with fever and systemic inflammation developed. Oral prednisolone (40 mg/day) rapidly improved symptoms and was tapered and replaced with etretinate (20 mg/day). Apremilast was subsequently introduced, and deucravacitinib was added on day 89, resulting in sustained complete remission.

Treatment with oral prednisolone at a dose of 40 mg/day was initiated, resulting in rapid resolution of fever and pustular lesions within several days. The dose of prednisolone was gradually tapered and subsequently replaced with etretinate (20 mg/day) for maintenance therapy (**Figure 2**).

### Follow-up and outcomes

After discharge, apremilast (30 mg twice daily) was introduced, followed by the addition of deucravacitinib (6 mg/day) on day 89 to further stabilize disease control. The combination regimen was well tolerated, and the patient has remained in complete clinical remission for two years without relapse or treatment-related adverse events (**Figure 2**).

## Discussion

Although various factors have been reported as triggers for GPP, particular attention has recently been drawn to SARS-CoV-2 infection. According to a PubMed-based review, including our present case, 11 cases of GPP exacerbation following SARS-CoV-2 infection and 10 cases following COVID-19 vaccination have been reported to date summarized in **Table 1** (4–20). Among the 10 vaccination-associated cases, seven occurred after BNT162b2 (Pfizer-BioNTech) vaccination, while one case each followed CoronaVac (Sinovac Biotech), BBIBP-CorV (Sinopharm), and mRNA-1273 (Moderna) vaccination. Interestingly, despite the higher frequency of systemic symptoms in post-vaccination cases, systemic corticosteroids were administered less frequently than in infection-related cases (40% vs. 73%). This apparent discrepancy likely reflects differences in treatment strategy rather than disease severity. In infection-related cases, corticosteroids were often prescribed not only to control acute systemic inflammation associated with GPP but also as part of the standard therapy for COVID-19 infection itself. Conversely, in post-vaccination cases, physicians may have been more cautious about systemic immunosuppression because of infection risk, and many patients had a prior history of biologic or retinoid therapy for psoriasis, facilitating the use of these agents instead. Collectively, these findings suggest that although both COVID-19 infection and vaccination can precipitate GPP flares, their clinical presentation, patient background, and management approaches differ considerably, likely reflecting distinct underlying immune activation pathways.

**Table 1:** The differences of GPP patients between Post-COVID infection and vaccine.

Parameter	Post–COVID-19 infection GPP	Post–COVID-19 vaccination GPP
Number of cases	11	10
Age range (years)	12–64 years (Mean 44.3)	18–72 years (Mean 47.3)
Sex (Male: Female)	5:6	5:5
History of psoriasis or GPP	6/11 (54.5%)	7/10 (78%)
Latency from trigger to onset (days)	0–28 days (Mean 18.9 days)	3–8 days (Mean 5 days)
Fever/malaise/arthralgia	4/11 (36%) ND in 3 cases	8/10 (80%) ND in 1 case
Systemic corticosteroid	8/11 (73%)	4/10 (40%)
Retinoid (etretinate, acitretin)	1/11 (9%)	4/10 (40%)
Cyclosporine	2/11 (18%)	3/10 (30%)
Biologics (adalimumab, infliximab, secukinumab, ixekizumab)	1/11 (9%)	6/10 (60%)

ND, Not described; GPP, Generalized pustular psoriasis; COVID-19, Coronavirus disease 2019.

Mechanistically, activation of the IL-36 receptor (IL-36R) signaling pathway plays a central role in the pathogenesis of GPP (1). IL-36 produced by various cells induces a cascade of pro-inflammatory cytokines and promotes neutrophilic infiltration in the epidermis (21). The SARS-CoV-2 spike protein (S protein) not only facilitates viral entry via ACE2 but also directly stimulates immune cells, leading to increased secretion of cytokines such as IL-6 and TNF-α (22). Given that both SARS-CoV-2 infection and vaccination can induce GPP flares, it is plausible that the shared spike protein acts as a common trigger, either directly or indirectly activating the IL-36 axis (23). Furthermore, recent studies have suggested that the S protein may interact with pattern-recognition receptors (PRRs) such as Toll-like receptors (TLRs) independent of ACE2 (24), thereby enhancing innate immune activation. This heightened inflammatory signaling, superimposed on a pre-existing psoriatic predisposition, could result in excessive IL-36R activation and subsequent disease exacerbation.

Collectively, these findings are consistent with a unifying immunopathogenic hypothesis in which both SARS-CoV-2 infection and vaccination may converge on shared inflammatory pathways, potentially involving spike protein–mediated activation of the IL-36 axis (23). The spike protein may engage TLR-mediated innate signaling on keratinocytes and immune cells, amplifying IL-36–dependent cytokine cascades and driving neutrophilic inflammation.

Our comparative analysis further revealed distinct clinical patterns between infection- and vaccine-associated GPP (**Table 1**). In infection-related cases, the latency from infection onset to GPP flare was longer (0–28 days, mean 18.9 days), whereas post-vaccination flares occurred more rapidly (3–8 days, mean 5 days). This difference likely reflects the contrasting kinetics of immune activation between natural infection and vaccination.

During SARS-CoV-2 infection, viral replication and antigen dissemination occur gradually, leading to sustained activation of innate immune sensors such as TLR3, TLR7, and STING, followed by delayed amplification of IL-1β, IL-36γ, TNF-α, and IL-17A pathways that drive neutrophilic inflammation (25, 26). In contrast, mRNA vaccination rapidly induces robust IL-1 and IL-6 (27, 28), triggering transient IL-36/Th17 activation in predisposed individuals. This acute and synchronized cytokine burst likely explains the shorter latency and self-limited nature of vaccine-related flares. Thus, the timing difference between infection- and vaccine-induced GPP flares may reflect distinct temporal patterns of innate and adaptive immune engagement.

In conclusion, this case suggests that SARS-CoV-2 infection may trigger GPP through an IL-36–related inflammatory pathway, based on clinical features and existing literature, although direct mechanistic evidence was not obtained in this patient.

### Patient perspective

The patient reported that the sudden flare of her skin disease following COVID-19 infection caused severe physical discomfort and emotional distress. She expressed relief after the rapid improvement of her symptoms and stated that long-term disease control has markedly improved her quality of life.

## Data Availability

The original contributions presented in the study are included in the article/supplementary material. Further inquiries can be directed to the corresponding author.
